# Supplementary material for: Pericytes regulate VEGF-induced endothelial sprouting through VEGFR1
Source: Nat Commun. 2017 Nov 17;8:1574. doi: 10.1038/s41467-017-01738-3 (PMC5691060; doi:10.1038/s41467-017-01738-3)
Supplement: Supplementary file 1 — Supplementary Information [file 41467_2017_1738_MOESM1_ESM.pdf]

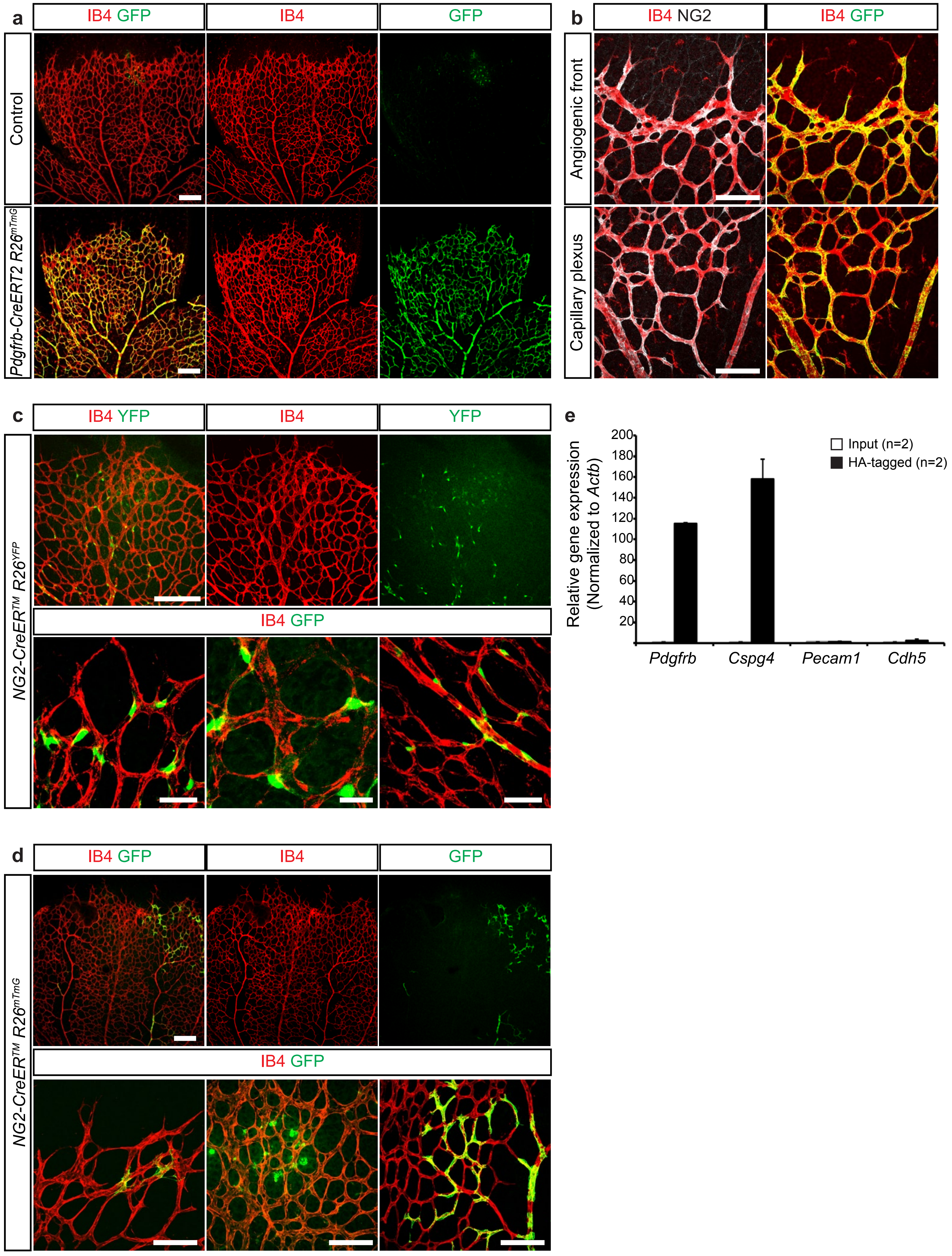
a
IB4 GFP
IB4
GFP
Control
Pdgfrb-CreERT2 R26mTmG
b
IB4 NG2
IB4 GFP
Angiogenic front
Capillary plexus
c
IB4 YFP
IB4
YFP
NG2-CreERTM R26YFP
IB4 GFP
d
IB4 GFP
IB4
GFP
NG2-CreERTM R26mTmG
IB4 GFP
e
Relative gene expression
(Normalized to Actb)
200
180
160
140
120
100
80
60
40
20
Input (n=2)
HA-tagged (n=2)
Pdgfrb
Cspg4
Pecam1
Cdh5

Supplementary Figure 1. Validation of *Pdgfrb-CreERT2* transgenic mice

a, *Pdgfrb-CreERT2*-mediated recombination (GFP expression, green) in retinal mural cells of P6 mice in the *Rosa26-mTmG* Cre reporter background. Note absence of GFP signal in Cre-negative littermate control. ECs, IB4 (red); scale bar, 200 µm.

b, Overlap of GFP (green) and NG2 (white) immunosignals in P6 *Pdgfrb-CreERT2 Rosa26-mTmG* double transgenic retinas.

c, **d**, Limited mural cell-specific GFP expression (green, **c**) and recombination outside the retinal vasculature (**d**) in *NG2-CreER*^{TM} *Rosa26-YFP* or *Rosa26-mTmG* double transgenic retinas from P6 pups. ECs, IB4 (red); scale bar, 200 µm (top rows in **c** and **d**), 50 µm (bottom row in **c**), 100 µm (bottom row in **d**).

e, Relative gene expression analysis by qPCR of *Pdgfrb*, *Cspg4*/*Ng2*, *Pecam1*, and *Cdh5* in P6 *Pdgfrb-CreERT2 Rpl22*$^{\mathrm{tm1.1Psam}}$ double transgenic animals after pull-down of HA-tagged Rpl22 ribosomal protein. Relative expression normalized to *Actb* and compared to input samples (value set as 1). Error bars, s.e.m.

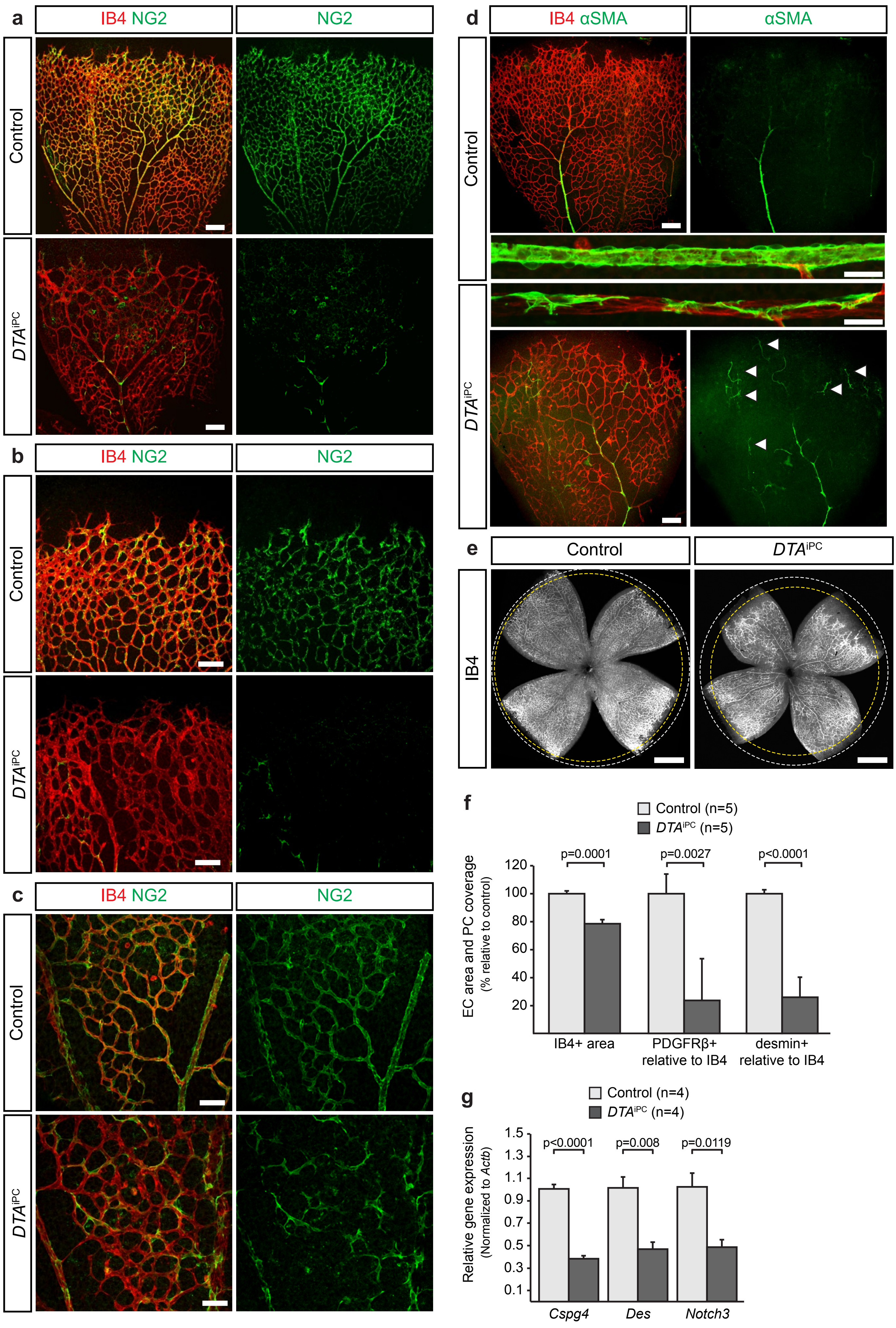
a
IB4 NG2
NG2
Control
DTA^iPC
b
IB4 NG2
NG2
Control
DTA^iPC
c
IB4 NG2
NG2
Control
DTA^iPC
d
IB4 αSMA
αSMA
Control
DTA^iPC
e
Control
DTA^iPC
IB4
f
Control (n=5)
DTA^iPC (n=5)
EC area and PC coverage
(% relative to control)
120
100
80
60
40
20
p=0.0001
p=0.0027
p<0.0001
IB4+ area
PDGFRβ+ relative to IB4
desmin+ relative to IB4
g
Control (n=4)
DTA^iPC (n=4)
Relative gene expression
(Normalized to Actb)
1.5
1.3
1.1
0.9
0.7
0.5
0.3
0.1
p<0.0001
p=0.008
p=0.0119
Cspg4
Des
Notch3

Supplementary Figure 2. Retinal vasculature after diphtheria toxin-mediated pericyte ablation

a, Confocal images showing reduction of NG2+ mural cells (green) in P6 *DTA*^{iPC} relative to littermate control retina whole-mounts. ECs, IB4 (red); scale bar, 150 µm.

b, **c**, Ablation of NG2+ pericytes (green) is more efficient in the P6 *DTA*^{iPC} peripheral retina (**b**) than in the central vasculature (**c**). ECs, IB4 (red); scale bar, 100 µm (**b**), 50 µm (**c**).

d, Reduction in arterial αSMA+ mural cell coverage in P6 *DTA*^{iPC} relative to littermate control retinas (scale bar, 150 µm). Note ectopic αSMA expression in the *DTA*^{iPC} peripheral retina (arrowheads). Images in the center show higher magnifications of retinal arteries (scale bar, 30 µm).

e, Isolectin B4-stained P7 control and *DTA*^{iPC} retina whole-mounts. Dashed circles indicate vessel-covered (yellow) and peripheral avascular areas (white), respectively. Scale bar, 500 µm.

f, Quantitation of the vascular area (IB4+), and pericyte coverage based on PDGFRβ+ or desmin+ area with respect to the IB4+ area in control and *DTA*^{iPC} P6 retinas. Error bars, s.e.m. p-values, Student's t-test.

g, Relative gene expression analysis by qPCR on whole retina lysates showing strong reduction in the expression of putative pericyte markers in *DTA*^{iPC} P6 retinas in comparison to control. Error bars, s.e.m. p-values, Student's t-test.

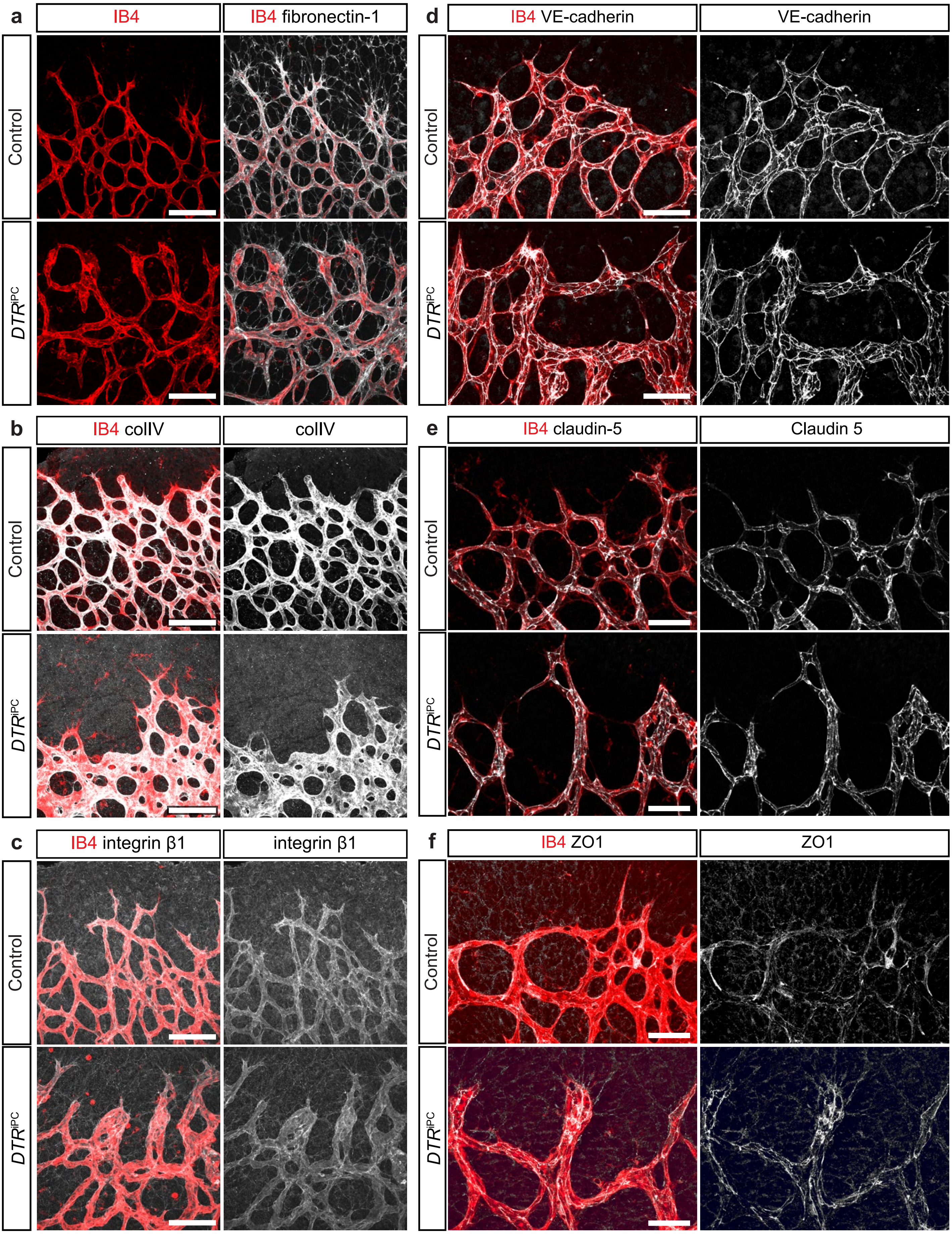

Supplementary Figure 3. ECM and junction protein expression after pericyte depletion

a-f, Confocal images showing IB4+ ECs (red) in *DTR*^{iPC} and control P6 retinas in combination with immunostaining (white) for fibronectin (FN1, **a**), collagen IV (colIV, **b**), integrin β1 (**c**), VE-cadherin (**d**), Claudin 5 (**e**), and ZO1 (**f**), as indicated. Scale bar, 100 μm (**a**-**c**), 50 μm (**d**-**f**).

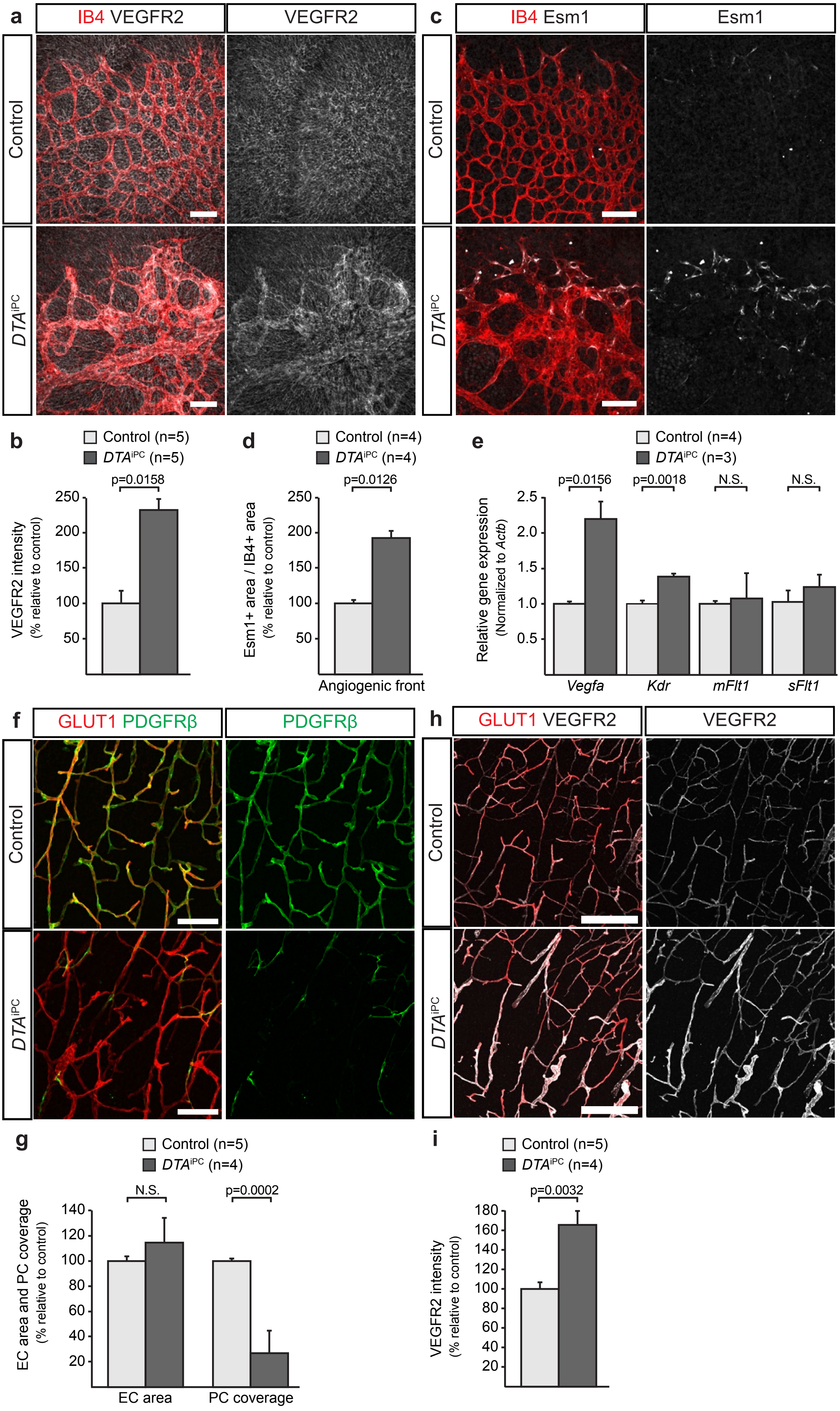

a
IB4 VEGFR2
VEGFR2
Control
DTA^iPC
c
IB4 Esm1
Esm1
Control
DTA^iPC
b
Control (n=5)
DTA^iPC (n=5)
p=0.0158
VEGFR2 intensity
(% relative to control)
250
200
150
100
50
d
Control (n=4)
DTA^iPC (n=4)
p=0.0126
Esm1+ area / IB4+ area
(% relative to control)
250
200
150
100
50
Angiogenic front
e
Control (n=4)
DTA^iPC (n=3)
p=0.0156
p=0.0018
N.S.
N.S.
Relative gene expression
(Normalized to Actb)
2.5
2.0
1.5
1.0
0.5
Vegfa
Kdr
mFlt1
sFlt1
f
GLUT1 PDGFRβ
PDGFRβ
Control
DTA^iPC
h
GLUT1 VEGFR2
VEGFR2
Control
DTA^iPC
g
Control (n=5)
DTA^iPC (n=4)
N.S.
p=0.0002
EC area and PC coverage
(% relative to control)
140
120
100
80
60
40
20
EC area
PC coverage
i
Control (n=5)
DTA^iPC (n=4)
p=0.0032
VEGFR2 intensity
(% relative to control)
180
160
140
120
100
80
60
40
20

Supplementary Figure 4. DTA-mediated pericyte ablation: molecular changes and effects on the brain vasculature.

a, Confocal images showing increased VEGFR2 (white) immunosignals in the peripheral plexus of P6 DTA^{iPC} retinas relative to littermate control. ECs, IB4 (red); scale bar, 100 µm.

b, Quantitation of VEGFR2 immunosignals intensity in the peripheral plexus of P6 DTA^{iPC} retinas relative to littermate control. Error bars, s.e.m. p-values, Student's t-test.

c, Confocal images showing increased Esm1+ (white) EC accumulation in the peripheral plexus of P6 DTA^{iPC} retinas relative to littermate control. ECs, IB4 (red); scale bar, 100 µm.

d, Quantitation of Esm1+ proportion with respect to total vascular area (IB4+) in the peripheral plexus of P6 DTA^{iPC} retinas relative to littermate control. Error bars, s.e.m. p-values, Student's t-test.

e, Relative gene expression analysis by qPCR on whole retina lysates showing strong induction of *Vegfa* expression in DTA^{iPC} P6 retinas in comparison to control. Error bars, s.e.m. p-values, Student's t-test. N.S., not statistically significant.

f, Confocal images showing reduction of PDGFRβ+ mural cells (green) in P6 DTA^{iPC} brain frontal cortex relative to littermate control. ECs, GLUT1 (red); scale bar, 100 µm.

g, Quantitation of EC area (GLUT1+) and pericyte coverage (PDGFRβ+ area with respect to GLUT1+ area) in the frontal brain cortex of P6 DTA^{iPC} mice relative to littermate control. Error bars, s.e.m. p-values, Student's t-test. N.S., not statistically significant.

h, Confocal images showing increased VEGFR2 immunosignals (white) in P6 DTA^{iPC} brain frontal cortex relative to littermate control. ECs, GLUT1 (red); scale bar, 200 µm.

i, Quantitation of VEGFR2 immunosignals intensity in the frontal brain cortex of P6 DTA^{iPC} mice relative to littermate control. Error bars, s.e.m. p-values, Student's t-test.

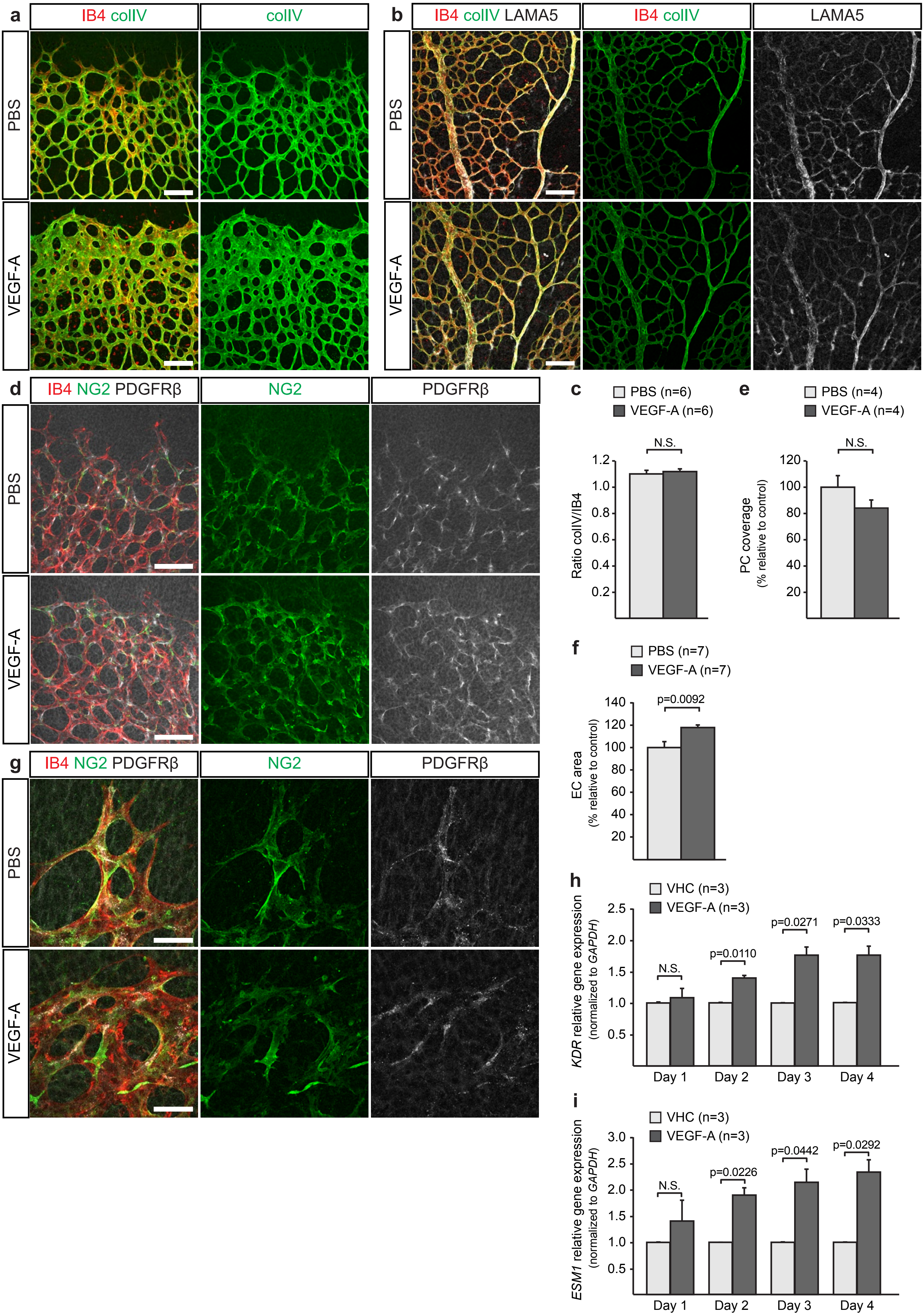

a
IB4 colIV
colIV
PBS
VEGF-A
b
IB4 colIV LAMA5
IB4 colIV
LAMA5
PBS
VEGF-A
d
IB4 NG2 PDGFRβ
NG2
PDGFRβ
PBS
VEGF-A
c
PBS (n=6)
VEGF-A (n=6)
N.S.
Ratio colIV/IB4
1.2
1.0
0.8
0.6
0.4
0.2
e
PBS (n=4)
VEGF-A (n=4)
N.S.
PC coverage
(% relative to control)
120
100
80
60
40
20
f
PBS (n=7)
VEGF-A (n=7)
p=0.0092
EC area
(% relative to control)
140
120
100
80
60
40
20
g
IB4 NG2 PDGFRβ
NG2
PDGFRβ
PBS
VEGF-A
h
VHC (n=3)
VEGF-A (n=3)
KDR relative gene expression
(normalized to GAPDH)
2.5
2.0
1.5
1.0
0.5
N.S.
p=0.0110
p=0.0271
p=0.0333
Day 1
Day 2
Day 3
Day 4
i
VHC (n=3)
VEGF-A (n=3)
ESM1 relative gene expression
(normalized to GAPDH)
3.0
2.5
2.0
1.5
1.0
0.5
N.S.
p=0.0226
p=0.0442
p=0.0292
Day 1
Day 2
Day 3
Day 4

Supplementary Figure 5. ECM composition and PC coverage analysis after VEGF-A injection

a-b, Maximum intensity projections of P6 retinas stained for IB4 (red), colIV (green) and laminin α5 (LAMA5, white) after intraocular injection of recombinant VEGF-A or vehicle control (PBS). Images in (**a**) show no changes in colIV deposition in the peripheral plexus, while the central plexus maintains a normal pattern of colIV and LAMA5 staining (**b**). Scale bar, 100 μm.

c, Quantitation of ECM deposition based on the analysis of the colIV+ area with respect to the vascular (IB4+) area in P6 retinas after intraocular injection of recombinant VEGF-A or vehicle control (PBS). Error bars, s.e.m. p-values, Student's t-test. N.S., not statistically significant.

d, Maximum intensity projections of P6 retinas stained for the pericyte markers NG2 (green) and PDGFRβ (white) after intraocular injection of recombinant VEGF-A or vehicle control (PBS). ECs, IB4 (red); scale bar, 100 μm.

e-f, Quantitation of PC coverage (**e**) and EC area (**f**) in P6 retinas after intraocular injection of recombinant VEGF-A or vehicle control (PBS). The slight decline in PC coverage is not significant and may reflect the increase in EC area as a consequence of vessel dilation. Error bars, s.e.m. p-values, Student's t-test. N.S., not statistically significant.

g, High-magnification confocal images of angiogenic sprouts in P6 retinas after intraocular injection of recombinant VEGF-A or vehicle control (PBS) highlighting the morphology and localization of PCs (NG2+ and PDGFRβ+, green and white, respectively) with respect to the ECs (IB4+, red); scale bar, 50 μm.

h, i, Relative gene expression analysis of *KDR* (**h**) and *ESM1* (**i**) by RT-qPCR in cultured HUVECs after stimulation with VEGF-A. Error bars, s.e.m. p-values, Student's t-test. N.S., not statistically significant.

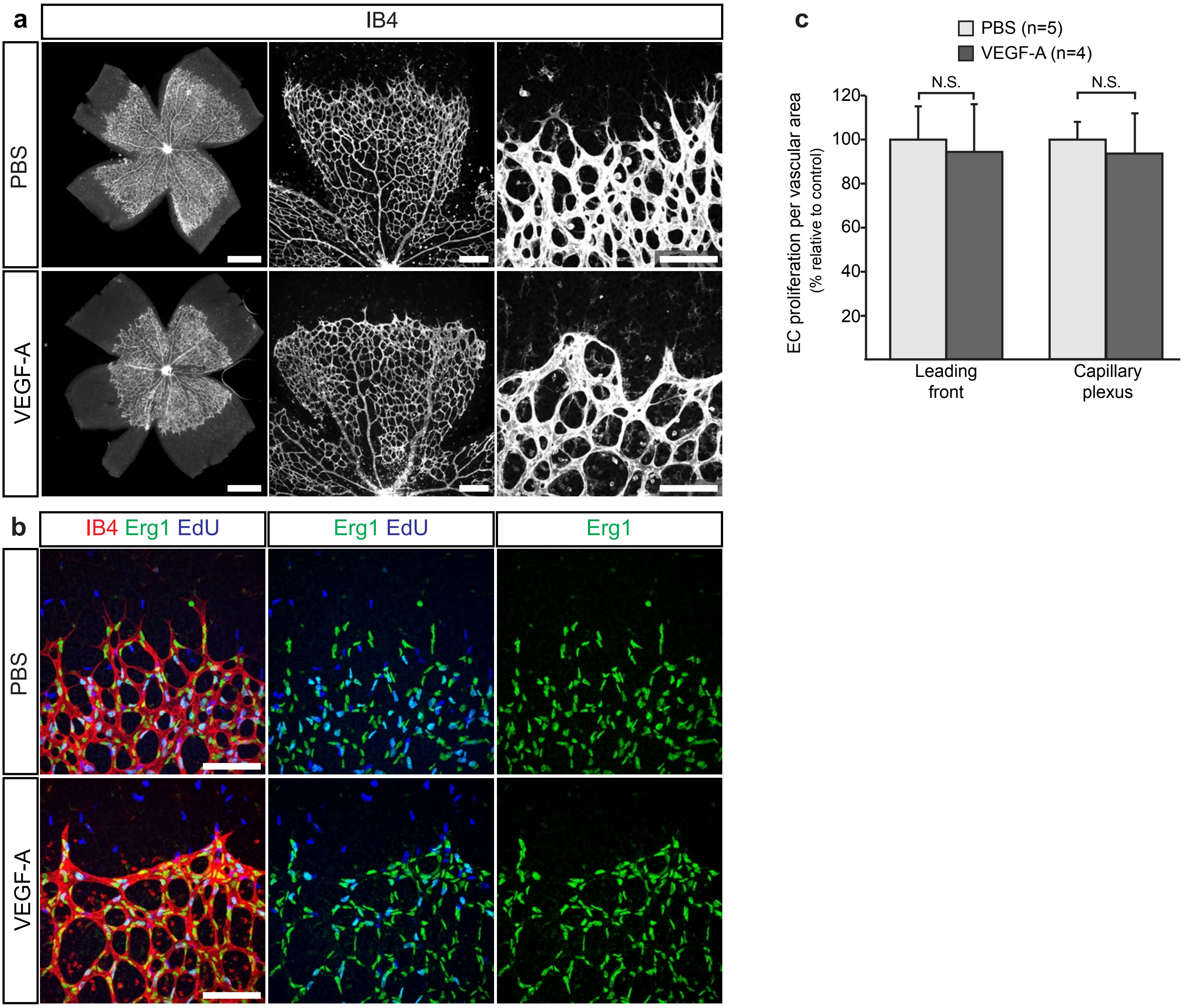

Supplementary Figure 6. Vascular alterations after VEGF-A injection

a, Appearance of IB4-stained retinal vessels after intraocular injection of recombinant VEGF-A or vehicle control (PBS) in P6 pups. Note that VEGF-A disrupts sprouting at the leading edge of the growing retinal vasculature. Scale bar, 500 μm (left), 200 μm (center) and 100 μm (right).

b, Confocal images of IB4 (red), Erg1 (green) and EdU (blue) labeled sprouts from P6 retinas highlighting the accumulation of EC nuclei at the front of the growing capillary bed. Scale bar, 100 μm.

c, Quantitation of EdU+ Erg1+ proliferating ECs at the angiogenic front and in the capillary plexus of P6 retinas. EC proliferation was not significantly changed within 4 hours after VEGF-A administration. Error bars, s.e.m. p-values, Student's t-test. N.S., not statistically significant.

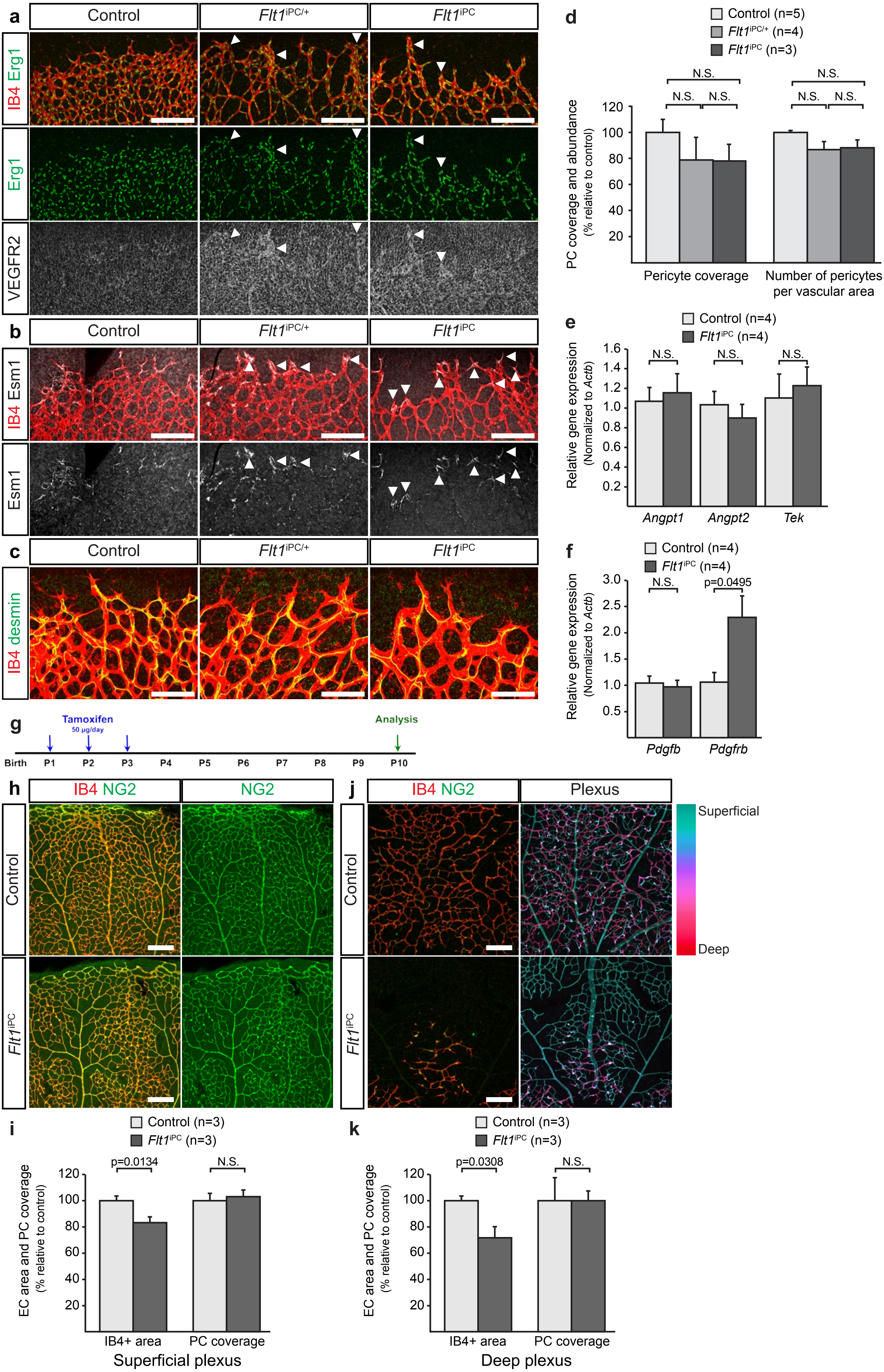

a
Control
Flt1iPC/+
Flt1iPC
IB4 Erg1
Erg1
VEGFR2
b
IB4 Esm1
Esm1
c
IB4 desmin
d
Control (n=5)
Flt1iPC/+ (n=4)
Flt1iPC (n=3)
N.S.
PC coverage and abundance (% relative to control)
Pericyte coverage
Number of pericytes per vascular area
e
Control (n=4)
Flt1iPC (n=4)
Relative gene expression (Normalized to Actb)
Angpt1
Angpt2
Tek
f
p=0.0495
Pdgfb
Pdgfrb
g
Tamoxifen
50 µg/day
Analysis
Birth
P1
P2
P3
P4
P5
P6
P7
P8
P9
P10
h
IB4 NG2
NG2
j
Plexus
Superficial
Deep
i
Control (n=3)
Flt1iPC (n=3)
p=0.0134
EC area and PC coverage (% relative to control)
IB4+ area
PC coverage
Superficial plexus
k
p=0.0308
Deep plexus

Supplementary Figure 7. Inactivation of *Flt1* in PDGFRβ+ cells

a, Confocal images of IB4 (red), Erg1 (green) and VEGFR2 (white) stained sprouts highlighting the accumulation of EC nuclei and enhanced VEGFR2 immunosignals (arrowheads) in *Flt1*$^{iPC/+}$ and *Flt1*^{iPC} P6 sprouts. Scale bar, 200 µm.

b, **c**, Esm1 (white) expression (arrowheads) in the IB4-stained (red) angiogenic front (**b**) and detection of desmin+ pericytes (**c**) in P6 control, *Flt1*$^{iPC/+}$ and *Flt1*^{iPC} retinas. Scale bar, 100 µm (**b**) and 200 µm (**c**).

d, Quantitation of pericyte coverage and of pericyte number (total desmin+ cells) normalized to IB4+ vascular area relative to control in P6 retinas. Error bars, s.e.m. p-values, one-way ANOVA. N.S., not statistically significant.

e, **f**, Relative gene expression analysis by qPCR in P6 whole retina lysates of relevant molecules involved in EC-pericyte communication through angiopoietins and Tie2 (**e**) and PDGF-B-PDGFRβ (**f**) signalling. Error bars, s.e.m. p-values, Student's t-test. N.S., not statistically significant.

g, Experimental scheme of tamoxifen administration for the generation of *Flt1*^{iPC} mutants and their analysis at P10.

h, Maximum intensity projections of the superficial vascular plexus in P10 retinas of control and *Flt1*^{iPC} mice stained for the pericyte marker NG2 (green) and IB4 (red); scale bar, 200 µm.

i, Quantitation of vascular area (IB4+ area) and PC coverage in the superficial plexus of control and *Flt1*^{iPC} P10 retinas. Error bars, s.e.m. p-values, Student's t-test. N.S., not statistically significant.

j, The first column shows maximum intensity projections of the deep vascular plexus in P10 retinas of control and *Flt1*^{iPC} mice stained for the pericyte marker NG2 (green) and IB4 (red). The second column shows a color-coded image comparing the superficial and deep vascular plexus in P10 retinas of control and *Flt1*^{iPC} mice; scale bar, 200 µm.

k, Quantitation of vascular area (IB4+ area) and PC coverage in the deep plexus of control and *Flt1*^{iPC} P10 retinas. Error bars, s.e.m. p-values, Student's t-test. N.S., not statistically significant.

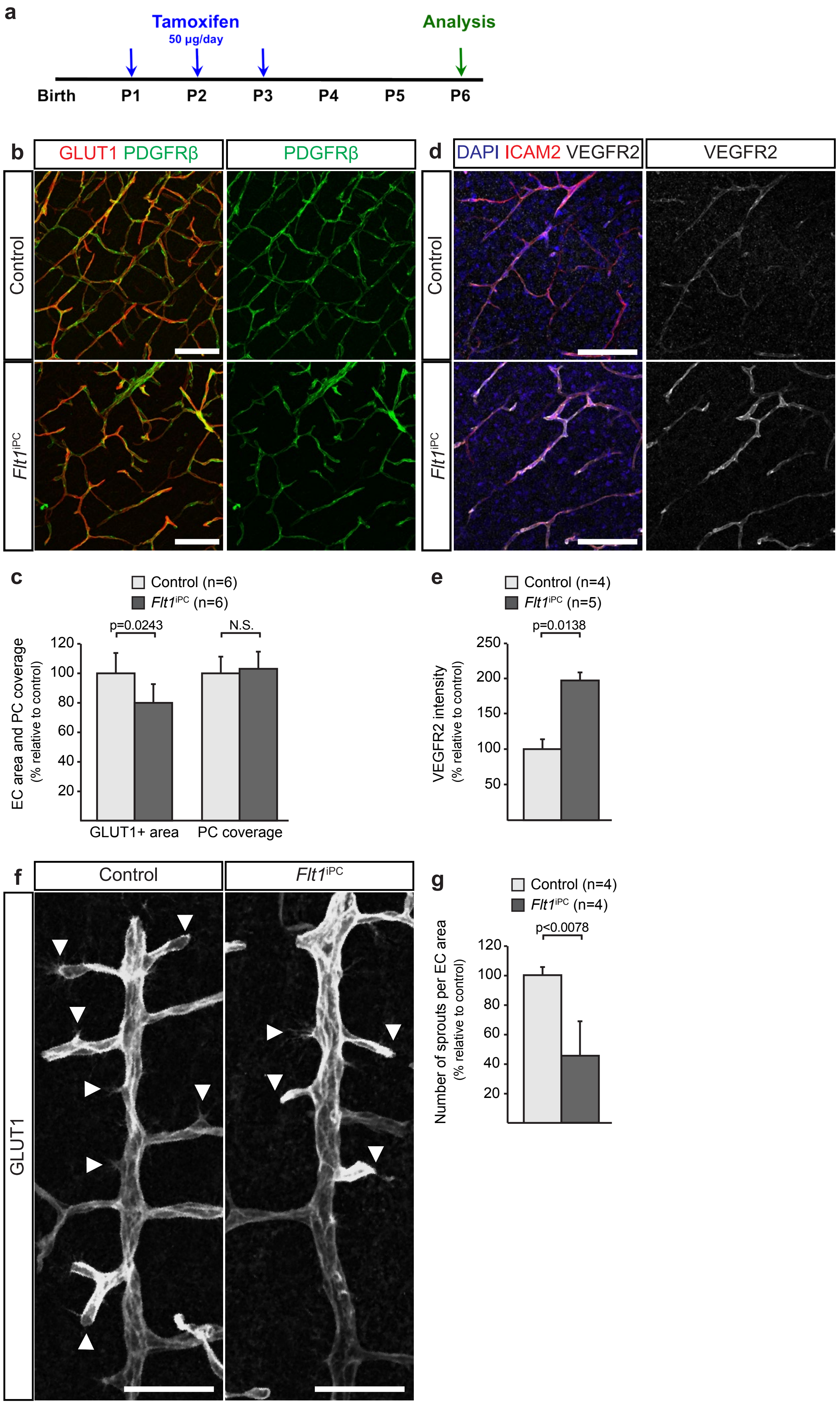
a
Tamoxifen
50 µg/day
Analysis
Birth
P1
P2
P3
P4
P5
P6
b
GLUT1 PDGFRβ
PDGFRβ
Control
Flt1iPC
c
Control (n=6)
Flt1iPC (n=6)
p=0.0243
N.S.
EC area and PC coverage
(% relative to control)
120
100
80
60
40
20
GLUT1+ area
PC coverage
d
DAPI ICAM2 VEGFR2
VEGFR2
Control
Flt1iPC
e
Control (n=4)
Flt1iPC (n=5)
p=0.0138
VEGFR2 intensity
(% relative to control)
250
200
150
100
50
f
Control
Flt1iPC
GLUT1
g
Control (n=4)
Flt1iPC (n=4)
p<0.0078
Number of sprouts per EC area
(% relative to control)
120
100
80
60
40
20

Supplementary Figure 8. Brain vascular phenotype of PC-specific *Flt1* mutants

a, Experimental scheme of tamoxifen administration for the generation of $Flt1^{iPC}$ mutants.

b, Maximum intensity projections of the frontal brain cortex vasculature of control and $Flt1^{iPC}$ P6 mice stained for the brain EC marker GLUT1 (red) and PDGFRβ (green); scale bar, 100 µm.

c, Quantitation of the vascular area (GLUT1+) and the PC coverage (PDGFRβ+ area normalized to the EC area) in the brain cortex of control and $Flt1^{iPC}$ P6 mice. Error bars, s.e.m. p-values, Student's t-test. N.S., not statistically significant.

d, Maximum intensity projections of the frontal brain cortex vasculature of control and $Flt1^{iPC}$ P6 mice stained for DAPI (blue), the EC luminal marker ICAM2 (red) and VEGFR2 (white); scale bar, 100 µm.

e, Quantitation of the VEGFR2 immunosignals intensity in the brain cortex of control and $Flt1^{iPC}$ P6 mice. Error bars, s.e.m. p-values, Student's t-test. N.S., not statistically significant.

f, High-magnification confocal images showing sprouting events (arrowheads) from brain cortex vessels in control and $Flt1^{iPC}$ P6 mice. ECs, GLUT1 (white); scale bar, 50 µm.

g, Quantitation of the number of sprouts (emerging vessels guided by a tip cell with visible filopodia) normalized to the EC area in the brain cortex of control and $Flt1^{iPC}$ P6 mice. Error bars, s.e.m. p-values, Student's t-test.

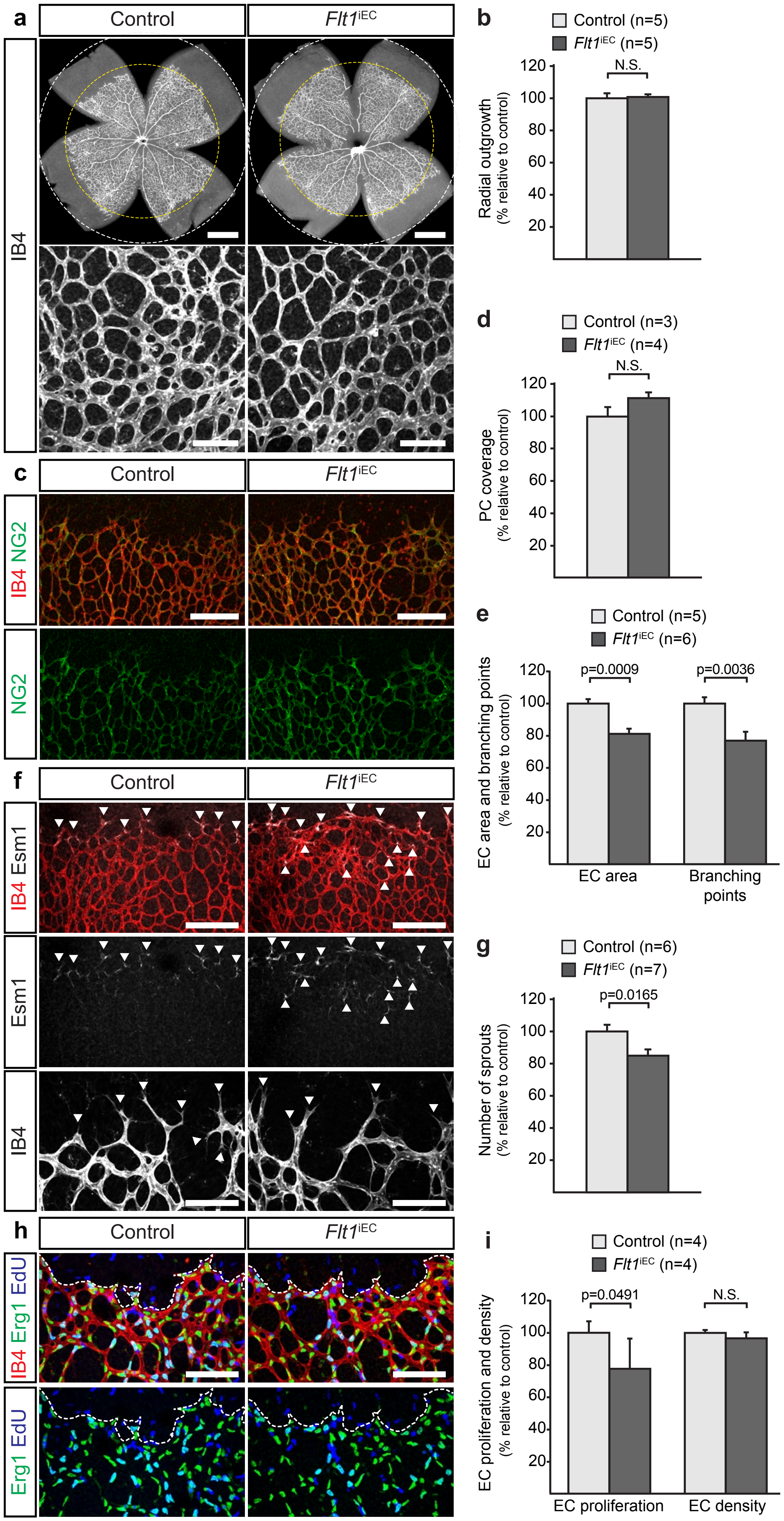

a
Control
Flt1iEC
IB4
b
Control (n=5)
Flt1iEC (n=5)
N.S.
Radial outgrowth
(% relative to control)
120
100
80
60
40
20
c
Control
Flt1iEC
IB4 NG2
NG2
d
Control (n=3)
Flt1iEC (n=4)
N.S.
PC coverage
(% relative to control)
e
Control (n=5)
Flt1iEC (n=6)
p=0.0009
p=0.0036
EC area and branching points
(% relative to control)
EC area
Branching points
f
Control
Flt1iEC
IB4 Esm1
Esm1
IB4
g
Control (n=6)
Flt1iEC (n=7)
p=0.0165
Number of sprouts
(% relative to control)
h
Control
Flt1iEC
IB4 Erg1 EdU
Erg1 EdU
i
Control (n=4)
Flt1iEC (n=4)
p=0.0491
N.S.
EC proliferation and density
(% relative to control)
EC proliferation
EC density

Supplementary Figure 9. Phenotype of EC-specific *Flt1* mutants

a, P6 control and *Flt1*^{iEC} retina whole-mounts stained with isolectin B4 (IB4). Dashed circles indicate vessel-covered (yellow) and peripheral avascular areas (white) in the low magnification overview pictures (top). Panels at the bottom show higher magnification of capillary plexus. Scale bar, 500 µm (top) and 100 µm (bottom).

b, Quantitation of radial outgrowth in the control and *Flt1*^{iEC} P6 retinal vasculature. Error bars, s.e.m. p-values, Student's t-test.

c, Maximum intensity projections of the frontal vascular plexus in P6 retinas of control and *Flt1*^{iEC} mice stained for the pericyte marker NG2 (green) and IB4 (red); scale bar, 200 µm.

d, Quantitation of PC coverage (NG2+ area normalized to IB4+ area) in the retinal vascular plexus of control and *Flt1*^{iEC} P6 retinas. Error bars, s.e.m. p-values, Student's t-test. N.S., not statistically significant.

e, Quantitation of the EC area (IB4+) and vessel branch points in the control and *Flt1*^{iEC} P6 retinal vasculature. Error bars, s.e.m. p-values, Student's t-test.

f, Esm1 (white) expression (arrowheads) in the IB4-stained (red/white) angiogenic front in P6 control and *Flt1*^{iEC} retinas. Scale bar, 200 µm (top, center) and 100 µm (bottom).

g, Quantitation of sprouts in the P6 control and *Flt1*^{iEC} vasculature. Error bars, s.e.m. p-values, Student's t-test.

h, Confocal images of IB4 (red), Erg1 (green) and EdU (blue) labeled angiogenic front in P6 control and *Flt1*^{iEC} mutants. Scale bar, 100 µm.

i, Quantitation of EC proliferation and the density of Erg1+ nuclei in the P6 control and *Flt1*^{iEC} vasculature. Error bars, s.e.m. p-values, Student's t-test.

Supplementary Table 1. Taqman probes used for qPCR interrogation of gene expression

Gene symbol	Target gene	Assay ID	Amplicon size	Label
Actb	β-actin	Mm00607939_s1	115	VIC
Angpt1	Angiopoietin 1	Mm00456503_m1	84	FAM
Angpt2	Angiopoietin 2	Mm00545822_m1	71	FAM
Cdh5	VE-cadherin	Mm00486938_m1	69	FAM
Cspg4	NG2 (chondroitin sulfate proteoglycan 4)	Mm00507257_m1	70	FAM
Des	Desmin	Mm00802455_m1	92	FAM
ESM1	Endothelial cell-specific molecule 1	Hs00199831_m1	93	FAM
Flt1 (mFlt1)	VEGFR1 (membrane-bound)	Mm00438994_m1	59	FAM
Flt1 (sFlt1)	VEGFR1 (soluble form)	No catalog item*	100	FAM
GAPDH	Glyceraldehyde 3-phosphate dehydrogenase	Hs99999905_m1	122	VIC
Kdr	VEGFR2	Mm01222419_m1	81	FAM
KDR	VEGFR2	Hs00911705_g1	84	FAM
Notch3	Notch3 receptor	Mm01345646_m1	83	FAM
Pdgfb	Platelet-derived growth factor B	Mm01298578_m1	88	FAM
Pdgfrb	Platelet-derived growth factor receptor β	Mm00435546_m1	61	FAM
Pecam1	CD31 (platelet and endothelial cell adhesion molecule 1)	Mm01242584_m1	71	FAM
Tek	Tie2	Mm00443254_m1	93	FAM
Vegfa	Vascular endothelial growth factor A	Mm00437306_m1	61	FAM
Vegfb	Vascular endothelial growth factor B	Mm00442102_m1	93	FAM
Vegfc	Vascular endothelial growth factor C	Mm01202432_m1	80	FAM

* Custom designed probe. Details of primers and probe sequence can be provided upon request.

NOTE: All probes used span exons. Additional specific details can be retrieved from http://www.thermofisher.com/order/genome-database/browse/gene-expression/
